# Guided chemotherapy based on patient-derived mini-xenograft models improves survival of gallbladder carcinoma patients

**DOI:** 10.1186/s40880-018-0318-8

**Published:** 2018-07-17

**Authors:** Ming Zhan, Rui-meng Yang, Hui Wang, Min He, Wei Chen, Sun-wang Xu, Lin-hua Yang, Qiang Liu, Man-mei Long, Jian Wang

**Affiliations:** 1 Department of Biliary-Pancreatic Surgery, Renji Hospital, School of Medicine, Shanghai Jiao Tong University, 160 Pujian Road, Shanghai, 200127 P. R. China; 2 Department of Pathology, Renji Hospital, School of Medicine, Shanghai Jiao Tong University, Shanghai, 200127 P. R. China; 3 Department of Pathology, Shanghai Ninth People’s Hospital, School of Medicine, Shanghai Jiao Tong University, Shanghai, 200011 P. R. China

**Keywords:** Gallbladder cancer, Mini-PDX, Chemosensitivity, Overall survival, Personalized therapy

## Abstract

**Background:**

Gallbladder carcinoma is highly aggressive and resistant to chemotherapy, with no consistent strategy to guide first line chemotherapy. However, patient-derived xenograft (PDX) model has been increasingly used as an effective model for in preclinical study of chemosensitivity.

**Methods:**

Mini-PDX model was established using freshly resected primary lesions from 12 patients with gallbladder to examine the sensitivity with five of the most commonly used chemotherapeutic agents, namely gemcitabine, oxaliplatin, 5-fluorouracil, nanoparticle albumin-bound (nab)-paclitaxel, and irinotecan. The results were used to guide the selection of chemotherapeutic agents for adjunctive treatment after the surgery. Kaplan–Meier method was used to compare overall survival (OS) and disease free survival (DFS) with 45 patients who received conventional chemotherapy with gemcitabine and oxaliplatin.

**Results:**

Cell viability assays based on mini-PDX model revealed significant heterogeneities in drug responsiveness. Kaplan–Meier analysis showed that patients in the PDX-guided chemotherapy group had significantly longer median OS (18.6 months; 95% CI 15.9–21.3 months) than patients in the conventional chemotherapy group (13.9 months; 95% CI 11.7–16.2 months) (*P* = 0.030; HR 3.18; 95% CI 1.47–6.91). Patients in the PDX-guided chemotherapy group also had significantly longer median DFS (17.6 months; 95% CI 14.5–20.6 months) than patients in the conventional chemotherapy group (12.0 months; 95% CI 9.7–14.4 months) (*P* = 0.014; HR 3.37; 95% CI 1.67–6.79).

**Conclusion:**

The use of mini-PDX model to guide selection of chemotherapeutic regimens could improve the outcome in patients with gallbladder carcinoma.

## Introduction

Gallbladder carcinoma, the most common biliary tract malignancy, is characterized by its aggressive growth and high lethality [1]. The disease generally carries a dismal prognosis due to its advanced stage at initial diagnosis and its recalcitrance to treatment [2, 3]. Despite advances in therapeutic strategies against gallbladder neoplastic disorders, surgical resection in combination with neoadjuvant or adjuvant therapies still remains the optimal treatment modality [4]. Unfortunately, only a small proportion of gallbladder carcinoma patients are eligible for surgical intervention. Despite the controversial role of adjuvant therapy, a multimodal therapeutic approach may benefit patients at high risk for recurrence, such as for those with lymph node metastasis or positive resection margins [5].

Recently, several new cytotoxic agents have been proven effective for advanced biliary tract cancer, with a reduction in the rate of morbidity and mortality [6]. A number of clinical trials are underway to examine the effectivity of adjuvant chemotherapy with gemcitabine, capecitabine, or S-1 in combination with platinum. Two phase III trials have shown that the combination of gemcitabine with cisplatin or oxaliplatin is superior to single-agent chemotherapy in improving the overall survival (OS) of biliary tract cancer patients and is now used as the standard palliative regimen [7]. Several phase II studies have also investigated the efficacy of targeted agents against EGFR, VEGF, HER2, and MEK [8].

Irinotecan is a camptothecin derivative that exerts antitumor activities against a variety of tumor types by targeting topoisomerase I, consequently leading to the formation of DNA double-strand breaks and inhibition of DNA synthesis. A retrospective study in patients with advanced biliary tract cancer suggested that the combination therapy of irinotecan with gemcitabine and fluorouracil confers promising survival benefits with manageable toxicities [9]. In addition, a phase II trial of gemcitabine in combination with irinotecan indicated comparable efficacy with historic control [10]. However, given the rarity of these tumors, evidence is still largely based on retrospective studies, surveillance, epidemiological, and end results database inquiries, single or multi-institutional prospective studies, and meta-analysis of studies on adjuvant therapy [11]. Therefore, systematic prospective investigations are urgently needed.

Continued efforts in gene expression profiling and genomic sequencing have uncovered the underlying complexity and molecular heterogeneity of gallbladder carcinoma, shedding light on the daunting challenges of therapeutic interventions [12]. The highly intrinsic heterogeneity of gallbladder carcinoma and varied responses to chemotherapeutic drugs mandate a personalized approach to gallbladder carcinoma treatment [13]. Recently, there have been increasing interests in the development and characterization of patient-derived xenograft (PDX) tumor models for cancer research [14]. PDX models retain the principal histologic and genetic characteristics of donor tumors and remain stable throughout passages. These models have been shown to be predictive of clinical outcomes and are used for preclinical drug evaluation, biomarker identification, biological studies, and personalized medicine strategies [15–17]. Thus, PDX models are useful in recapitulating the complexity and heterogeneity of gallbladder carcinoma. However, there are limitations for direct application of traditional PDX models on gallbladder carcinoma patients. It typically takes 4–8 months for an established PDX model to be ready for assessing drug sensitivity, which is unduly long for initiation of drug-sensitivity guided treatment of gallbladder carcinoma patients. Mini-PDX model offers an effective alternative as it only takes about 7 days for the model to complete drug sensitivity test and could thus provide guidance for prompt personalized selection of individual drugs for each patient.

In the present study, we first established a mini-PDX model using fresh primary tumor cells from 12 surgically resected gallbladder carcinomas. Responses to five of the most commonly used chemotherapeutic agents including gemcitabine, oxaliplatin, 5-fluorouracil, nanoparticle albumin-bound (nab)-paclitaxel, and irinotecan were examined. We treated 12 gallbladder carcinoma patients with the top two efficient agents as obtained from the results of drug sensitivity tests. Patient outcomes then were compared with 45 gallbladder carcinoma patients who were conventionally treated with gemcitabine and oxaliplatin. Our results demonstrated that drug sensitivity-guided chemotherapy yielded significantly better outcomes as revealed by increased OS and disease free survival rate (DFS) than conventional chemotherapy. Analysis of the clinicopathologic features of gallbladder carcinoma patients further revealed that gemcitabine sensitivity was associated with nerve invasion while irinotecan sensitivity was associated with tumor size, lymph node metastasis and TNM stage. Lastly, we also found strong association between responses to drug sensitivity-guided therapy and the expression of several important chemoresistance-related proteins (e.g., p53 and *P*-gp).

## Materials and methods

### Tissue specimen acquisition

Fresh tissue specimens were obtained from 57 treatment naïve gallbladder carcinoma patients who underwent surgery at the Department of Biliary-Pancreatic Surgery of Renji Hospital, School of Medicine, Shanghai Jiao Tong University between September 2014 and September 2016. Gallbladder carcinoma was staged according to the TNM classification (AJCC 7th edition).

### Follow-up

Follow-ups were conducted once every month during the first year post surgery and once every 3 months thereafter. Phone calls were made to patients and their relatives according to the follow-up guidelines of the National Comprehensive Cancer Network of China. OS was calculated from the date of surgery until the date of the final follow-up visit or death and DFS was calculated from the date of surgery until the final follow-up visit or tumor recurrence. The final follow-up visit was September 2017.

### Mini-PDX models and drug sensitivity assays

Mini-PDX models were established using freshly removed gallbladder carcinoma tissues from 12 patients. Drug sensitivity was examined using the OncoVee™-Mini PDX assay (LIDE Biotech, Shanghai, China). Briefly, gallbladder carcinoma samples were washed with Hank’s balanced salt solution (HBSS) to remove non-tumor tissues and necrotic tumor tissues. After morselization, the tumor tissues were digested with collagenase at 37 °C for 1–2 h. Cells were collected followed by removal of blood cells and fibroblasts. Then, gallbladder carcinoma cell suspension was transferred to the HBSS washed capsules.

Four-weeks-old BALB/c nude mice (SLARC Inc., Shanghai, China), weighing 15–20 g each, were used for subcutaneous implantation. A small skin incision was made and the capsule was embedded in the subcutaneous tissue. Generally, each mouse received 3 capsules. Drugs (gemcitabine, 60 mg/kg, IP, Q4D × 2; oxaliplatin, 5 mg/kg, IP, Q4D × 2; 5-fluorouracil, 25 mg/kg, IP, QD × 5; nab-paclitaxel, 20 mg/kg, IV, QD × 5; irinotecan, 50 mg/kg, IP, Q4D × 2) were administered for 7 days respectively. Normal saline was used as the control. Anti-tumor activity was evaluated based on the relative fluorescence units (RFU) using the CellTiter-Glo^®^ Luminescent Cell Viability Assay (Promega, Madison, WI, USA). Proliferation rate was calculated using the equation:

$$ {\text{Proliferation rate}} = {{\left( {{\text{RFU}}^{{{\text{D}}7}}  - {\text{RFU}}^{{{\text{D}}0}} } \right)_{{{\text{drug}}}} } \mathord{\left/ {\vphantom {{\left( {{\text{RFU}}^{{{\text{D}}7}}  - {\text{RFU}}^{{{\text{D}}0}} } \right)_{{{\text{drug}}}} } {\left( {{\text{RFU}}^{{{\text{D}}7}}  - {\text{RFU}}^{{{\text{D}}0}} } \right)_{{{\text{placebo}}}} }}} \right. \kern-\nulldelimiterspace} {\left( {{\text{RFU}}^{{{\text{D}}7}}  - {\text{RFU}}^{{{\text{D}}0}} } \right)_{{{\text{placebo}}}} }} $$

**Fig. 1:**
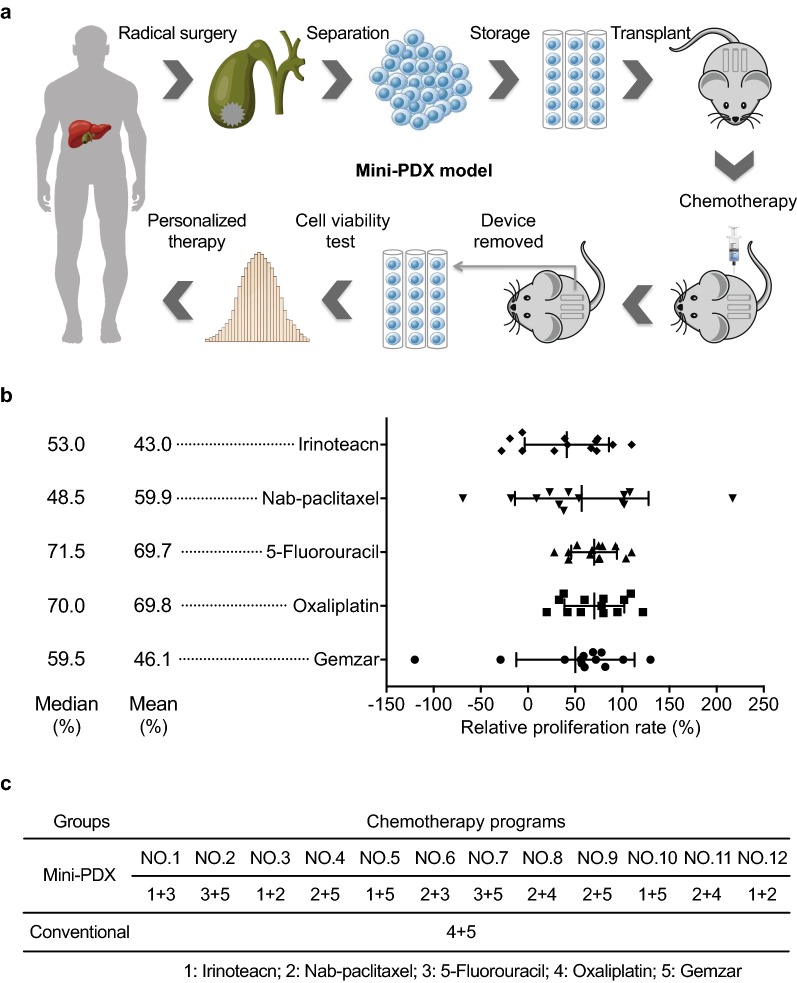
An overview of the generation of the mini-PDX model. **a** Gallbladder carcinoma cells from gallbladder carcinoma patients under surgical resection were transferred to the HBSS washed capsules and then subcutaneously implanted in BALB/c nude mice. Drugs or placebo (saline) were injected via the tail veins or intraperitoneally. Finally, the capsules were taken out and the anti-tumor activity was evaluated by detecting cell viabilities via CTG assays. Based on the anti-tumor activity data of the mini-PDX models, the optimal chemotherapy regimens were selected for different gallbladder carcinoma patients. **b** Scatter plot shows the results of the relative proliferation rate of the five drugs tested on the mini-PDX model among the 12 gallbladder carcinoma patients. **c** Detailed results reveal the two most effective agents chosen for treating the patients in the mini-PDX group and the conventional chemotherapy group

The study flowchart is shown in Fig. 1a. All procedures were performed under specific pathogen free conditions and carried out in accordance with the guidelines for the Care and Use of Laboratory Animals of the National Institutes of Health.

### Immunohistochemistry

Formalin-fixed, paraffin-embedded tissues were immunohistochemically stained as described previously [18]. Primary antibodies against the following proteins were used: p53 (1:200, ab1101, Abcam, Cambridge, UK), Ki-67 (1:600, ab15580, Abcam), *P*-gp (1:800, 13978, CST, MA, USA), MRP1 (1:200, 72202, CST), Bcl2 (1:400, 15071, CST), TS (1:100, ab58287, Abcam), GST-π (1:200, ab58287, Abcam), and Bcl-2 (1:400, 12286, CST). Immunohistochemical staining was semi-quantitatively scored by rating staining intensity of a protein of interest (I: negative, 0; weak, 1; moderate, 2; intense, 3) and the percentage of positively stained cells (P: 0%–5%, scored 0; 6%–35%, scored 1; 36%–70%, scored 2; and > 70%, scored 3) to obtain a final score (Q), which was defined as the product of I × P. Two senior pathologists evaluated the tissues independently in a blinded manner.

### Statistical analysis

Data are presented as mean ± standard deviation (SD). Normally distributed continuous variables were analyzed using unpaired Student’s *t-*test. For multiple comparisons, the Tukey–Kramer honestly significant difference test was applied following ANOVA. Kaplan–Meier method and log-rank test were used to analyze OS and DFS. Data were censored for patients who were lost to follow-up. Pearson χ^2^ test was used to analyze the correlation between clinicopathological variables and drug sensitivity. SPSS 17.0 software (SPSS Inc., Chicago, IL, USA) was used for all statistical analyses. For all analysis, *P* < 0.05 was considered statistically significant.

### Ethical approval

The present study was approved by the Ethical Committee of Renji Hospital, School of Medicine, Shanghai Jiao Tong University. Written informed consents were provided by all participants before enrollment. All procedures were performed in accordance with the Ethical Standards of Institutional/National Research Committees and the 1964 Helsinki Declaration, its later amendments, or similar ethical standards.

## Results

### The mini-PDX model yields drug sensitivity patterns of gallbladder carcinoma patients

Cell viability assays showed that the mean proliferation rate of gallbladder carcinoma cells treated with gemcitabine, oxaliplatin, 5-fluorouracil, nab-paclitaxel, or irinotecan was 46.1%, 69.8%, 69.7%, 59.9%, and 43.0%, respectively (Fig. 1b). Drug sensitivity varied substantially, with the highest relative proliferation rate at 110% and the lowest at 28% of irinotecan implying the requirement of individualized therapy (Fig. 1b). The agents used in the investigated patients are shown in Fig. 1c.

### Mini-PDX-guided chemotherapy is superior to conventional chemotherapy in prolonging survival of gallbladder carcinoma patients

**Fig. 2:**
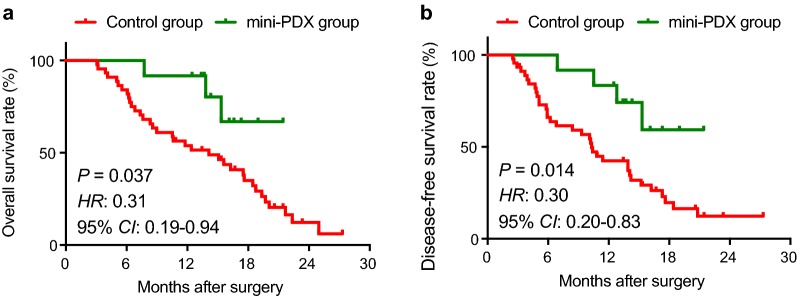
Comparison of the prognosis of gallbladder carcinoma patients between the conventional chemotherapy group and the mini-PDX-guided chemotherapy group. The 12 gallbladder carcinoma patients who received agents based on the mini-PDX results had higher overall survival (**a**) and disease-free survival rates (**b**) than the 45 gallbladder carcinoma patients treated with conventional chemotherapeutic drugs. **b** The two curves were compared using log-rank test

The cohort included 24 males and 33 females with a mean age 66.6 ± 9.5 years. Patients in the PDX-guided chemotherapy group included 5 male (41.7%) and 7 female patients (58.3%) with a median age of 67 years (range 56–87 years). The PDX-guided chemotherapy group and the conventional chemotherapy group had comparable demographic and baseline characteristics (Table 1). Kaplan–Meier analysis showed that patients in the PDX-guided chemotherapy group had significantly longer median OS (18.6 months; 95% CI 15.9–21.3 months) than patients in the conventional chemotherapy group (13.9 months; 95% CI 11.7–16.2 months) (*P* = 0.030; HR 3.18; 95% CI 1.47–6.91) (Fig. 2a). Patients in PDX-guided chemotherapy group also had significantly longer median DFS (17.6 months; 95% CI 14.5–20.6 months) than patients in the conventional chemotherapy group (12.0 months; 95% CI 9.7–14.4 months) (*P* = 0.014; HR 3.37; 95% CI 1.67–6.79) (Fig. 2b).

**Table 1:** Patient demographic and baseline characteristics

Characteristic	All	Conventional chemotherapy	PDX-guided chemotherapy	OR (95% CI)	*P* ^a^
No.	57	45	12		
Female gender, n (%)	33 (57.89)	26 (57.78)	7 (58.33)	1.02 (0.28–3.72)	0.972
Age, years, n (%)					
< 65	23 (40.35)	18 (40)	5 (41.67)	1.07 (0.29–3.91)	0.917
Gallstone, n (%)					
Yes	44 (77.19)	36 (80)	8 (66.67)	0.5 (0.12–2.04)	0.555
CA19-9, U/mL, n (%)					
< 37	16 (28.07)	13 (28.89)	3 (25)	0.82 (0.19–3.52)	0.924
Tumor size, cm, n (%)					
< 4	37 (64.91)	31 (68.89)	6 (50)	0.45 (0.12–1.65)	0.223
Tumor differentiation, n (%)					
Well and moderate	31 (54.39)	23 (51.11)	8 (66.67)	1.91 (0.5–7.27)	0.525
Nerve invasion, n (%)					
Yes	21 (36.84)	15 (33.33)	6 (50)	0.5 (0.14–1.82)	0.288
Lymph node metastasis, n (%)					
Yes	21 (36.84)	14 (31.11)	7 (58.33)	0.4 (0.1–1.66)	0.375
^b^TNM stage, n (%)					
IIIA	36 (63.16)	31 (68.89)	5 (41.67)	0.32 (0.09–1.2)	0.082
IIIB and IV	21 (36.84)	14 (31.11)	7 (58.33)		

*PDX* patient-derived xenograft

^a^Chi square test

^b^Tumor stage was defined according to the American Joint Committee on Cancer (AJCC) TNM staging system (AJCC 7th edition)

### Correlation between drug sensitivity and clinicopathological variables and biomarkers

**Fig. 3:**
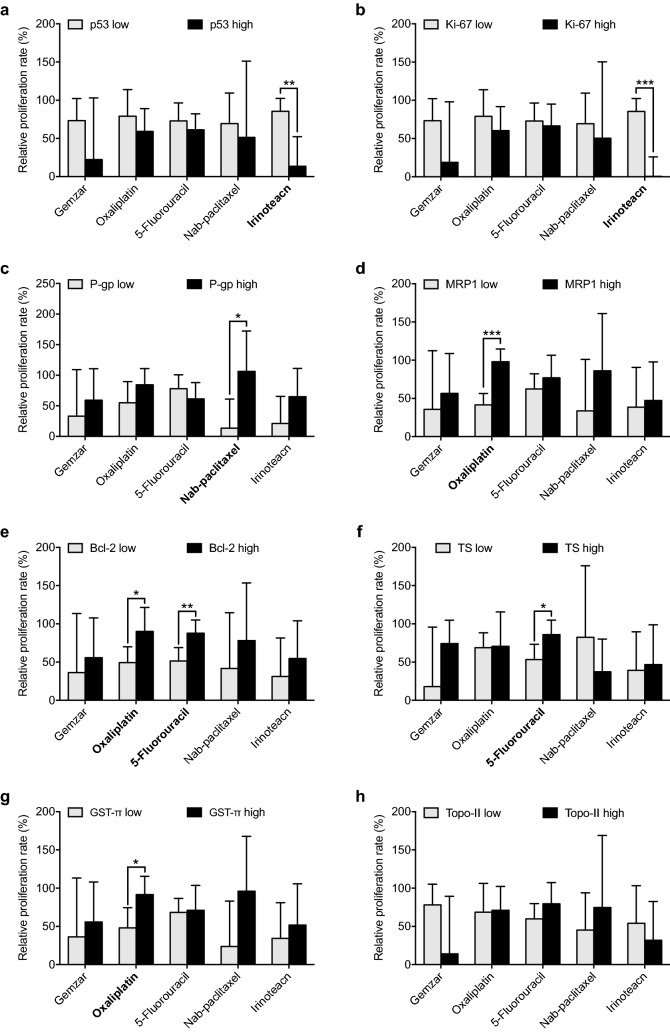
Correlation between drug sensitivity and various biomarkers. The relationship between the efficacy of the five drugs (gemcitabine, oxaliplatin, 5-fluorouracil, nab-paclitaxel, and irinotecan) and p53 (**a**), Ki-67 (**b**), *P*-gp (**c**), MRP1 (**d**), Bcl-2 (**e**), TS (**f**), GST (**g**) as well as TOP II (**h**) expressions in gallbladder carcinoma patients. Error bar, SD, *P < 0.05; **P < 0.01; ***P < 0.001; Student’s *t* test (two-tailed)

Our correlation analysis revealed that gemcitabine sensitivity was correlated with nerve invasion, while irinotecan efficacy was correlated with tumor size, lymph node metastasis and TNM stage (Table 2). In our mini-PDX models, irinotecan exhibited significantly greater cytotoxic effects on gallbladder carcinoma cells with high p53 or Ki-67 expression versus those with low p53 or Ki-67 expression (Fig. 3a, b). Furthermore, nab-paclitaxel demonstrated significantly greater anti-tumor effects on gallbladder carcinoma cells with low *P*-gp expression versus those with high *P*-gp expression (Fig. 3c). Oxaliplatin demonstrated significantly greater inhibitory effects on gallbladder carcinoma cells with high MRP1, Bcl-2 or GST-π expression; in contrast, 5-fluorouracil exhibited significantly greater inhibitory effects on gallbladder carcinoma cells with low Bcl-2 or TS expression (Fig. 3d–g).

**Table 2:** Association analysis between clinicopathologic characteristics and chemosensitivity

Proliferation rate (%)	Gemcitabine	*P*	Oxaliplatin	*P*	5-Fluorouracil	*P*	Nab-paclitaxel	*P*	Irinotecan	*P*
Tumor size, cm										
< 4	65.3 ± 11.5	0.316	72.2 ± 27.9	0.814	66.5 ± 30.0	0.684	84.8 ± 28.5	0.257	71.0 ± 26.4	*0.049*
≥ 4	26.8 ± 88.6		67.3 ± 40.2		72.8 ± 21.7		35.0 ± 97.4		15.0 ± 51.9	
Tumor differentiation										
Well and moderate	38.5 ± 78.2	0.699	55.0 ± 28.7	0.127	61.7 ± 24.6	0.292	35.3 ± 67.5	0.264	31.7 ± 46.8	0.449
Poor	53.7 ± 51.2		84.5 ± 32.6		77.7 ± 25.2		84.5 ± 76.1		54.3 ± 52.7	
Nerve invasion										
No	10.3 ± 72.1	*0.044*	74.8 ± 26.8	0.618	71.2 ± 30.2	0.848	82.7 ± 94.2	0.303	37.2 ± 56.5	0.700
Yes	81.8 ± 24.8		64.7 ± 40.4		68.2 ± 21.9		37.2 ± 40.9		48.8 ± 44.8	
Lymph node metastasis										
No	30.2 ± 85.7	0.491	55.4 ± 34.3	0.219	69.8 ± 34.1	0.989	21.4 ± 66.3	0.128	7.4 ± 38.5	*0.024*
Yes	57.4 ± 46.3		80.0 ± 30.5		69.6 ± 19.7		87.4 ± 69.1		68.4 ± 40.0	
TNM stage										
I–IIIA	18.3 ± 94.0	0.305	61.0 ± 36.9	0.543	64.0 ± 36.4	0.606	13.3 ± 73.6	0.122	− 7.5 ± 22.2	*0.004*
IIIB–IV	60.0 ± 43.5		74.1 ± 32.7		72.5 ± 20.0		83.3 ± 65.1		68.3 ± 37.0	

*P* values were calculated by unpaired *t*-test (2-sided)

## Discussion

Cancer drug development has been hampered by a lack of preclinical models that could reliably predict the efficacy of novel compounds in cancer patients. PDX models have gained more popularity in the past several years over conventional models in predicting postoperative chemosensitivity of various tumors [19]. Here, we present the first piece of preclinical and clinical evidence for the utility of optimized mini-PDX model in guiding adjuvant chemotherapy of gallbladder cancer patients. Moreover, when prospectively comparing the efficacy of PDX-guided chemotherapy versus conventional chemotherapy, we found that PDX-guided chemotherapy significantly improved the survival outcome of gallbladder carcinoma patients. Additionally, the correlations between some clinicopathological features, biomarkers and drug efficacy were also analyzed. Our present study showed that the mini-PDX models well-recapitulated the tumor behaviors of gallbladder carcinoma patients and could provide important guidance for oncologists in making informed decision on individualized chemotherapy.

Most translational cancer studies require effective preclinical models [20–23]. Human cancer models for drug screening started in the 1970s with the help of conventional cell lines. Although convenient and easy to use, these cell lines-based studies lack the predictive value of specific cancer types for clinical application. The preclinical PDX models has circumvent the limitations of conventional cell line-based models and are now more commonly used. These models can provide drug sensitivities that mimic the clinical response of cancer patients to cytotoxic agents [24]. Also, since PDX models correlate well with the pathologic characteristics and genetic features of the tumors of individual patients, they are becoming the preferred preclinical tool to improve the drug development process [24]. However, the long time in establishing PDX models restrains their usage in more aggressive cancers like gallbladder carcinoma; not to mention that several transplantation cycles are needed for tumor xenograft formation in PDX models and which might alter the properties of the originally transplanted tumor. Reports have demonstrated that the finally formed tumor xenograft is subsequently a more aggressive phenotype and behaves more like metastatic tumor [25]. Most gallbladder carcinoma patients are diagnosed at advanced stages, and thus require prompt initiation of anti-tumor therapy. In this perspective, mini-PDX model is the more suitable alternative.

Currently, gemcitabine, oxaliplatin, 5-fluorouracil, nab-paclitaxel, and irinotecan are the five most potentially effective agents for gallbladder carcinoma patients [26]. Single or combination chemotherapies of these drugs have shown improved median survival rates of gallbladder carcinoma patients. However, further clinical application of these drugs are often impeded by the uncommon nature of gallbladder carcinoma and efficacy data are mostly based on studies from other biliary tract tumors like intrahepatic or extrahepatic cholangiocarcinoma, which are now considered to be different from gallbladder carcinoma [12, 27]. In this study, an improved prognosis was observed when the effectiveness of five chemotherapeutic drugs were separately tested using mini-PDX models, after which the two most effective drugs identified were prospectively prescribed to gallbladder carcinoma patients. This personalized treatment provides a scientific rationale for clinical therapy and avoids the side effects from clinical experience-guided medication. Aside from cytotoxic chemotherapeutic agents, several targeted agents against EGFR, VEGF or MEK have also been reported to be useful in treating gallbladder cancer. Therefore, our mini-PDX model could be suitable for preclinical testing of the effectiveness of these drugs in the coming future.

Aside from providing reliable references for the choice of drugs clinically, the mini-PDX models, when combined with other technical methods, could also accelerate the understanding of the underlying mechanisms of oncogenesis, tumor progression and chemoresistance. In this present study, we have found that gemcitabine sensitivity was correlated with nerve invasion, and irinotecan efficacy was associated with tumor size, lymph node metastasis and TNM stage. Future studies with a larger sample set are required to confirm these relationships and help elucidate the underlying mechanisms. P53, Ki-67, *P*-gp, MRP1, Bcl-2, TS, GST-π and Topo-II are important in modulating chemosensitivity [28, 29]. The reactivity to irinotecan in our mini-PDX model was found to correlate with p53 and Ki-67 expression in gallbladder carcinoma patients. Oxaliplatin sensitivity was associated with MRP1 and Bcl-2 expression. These findings demonstrate that the mini-PDX model is effective in predicting chemosensitivity of gallbladder carcinoma patients and lends support to the future application of this method in clinical practice. In addition, based on our obtained results, chemo resistant and chemosensitive PDX tumor models could be developed to study the molecular mechanisms of chemoresistance in gallbladder carcinoma, which would be conducive in finding potential therapeutic targets and predictive markers of chemosensitivity for gallbladder cancer patients. Hence, mini-PDX models has potential prospect of being extended in the treatment of other types of malignant tumors.

## Conclusions

Our results show that the mini-PDX model is an effective tool in guiding the choice of chemotherapeutic regimens for gallbladder carcinoma patients and that PDX-guided chemotherapy can significantly improve the survival of gallbladder carcinoma patients compared to conventional chemotherapy. However, further confirmations from studies with larger sample size are needed.

## Abbreviations

PDX: patient-derived xenograft
OS: overall survival
DFS: disease free survival rate
HBSS: Hank’s balanced salt solution
RFU: relative fluorescence units
SD: standard deviation
